# An RNA pseudoknot mediates toxin translation and antitoxin inhibition

**DOI:** 10.1073/pnas.2403063121

**Published:** 2024-06-27

**Authors:** Athina Eleftheraki, Erik Holmqvist

**Affiliations:** ^a^Department of Cell and Molecular Biology, Biomedical Center, Uppsala University, Uppsala 75124, Sweden; ^b^Uppsala Antibiotic Center, Uppsala University, Uppsala 75123, Sweden

**Keywords:** toxin–antitoxin, translation control, small RNA, posttranscriptional regulation, RNA structure

## Abstract

Type I toxin–antitoxin systems (T1TAs) are bipartite bacterial loci encoding a growth-inhibitory toxin and an antitoxin small RNA (sRNA). In many of these systems, the transcribed toxin mRNA is translationally inactive, but becomes translation-competent upon ribonucleolytic processing. The antitoxin sRNA targets the processed mRNA to inhibit its translation. This two-level control mechanism prevents cotranscriptional translation of the toxin and allows its synthesis only when the antitoxin is absent. Contrary to this, we found that the *timP* mRNA of the *timPR* T1TA locus does not undergo enzymatic processing. Instead, the full-length *timP* transcript is *both* translationally active and can be targeted by the antitoxin TimR. Thus, tight control in this system relies on a noncanonical mechanism. Based on the results from in vitro binding assays, RNA structure probing, and cell-free translation experiments, we suggest that *timP* mRNA adopts mutually exclusive structural conformations. The active form uniquely possesses an RNA pseudoknot structure which is essential for translation initiation. TimR preferentially binds to the active conformation, which leads to pseudoknot destabilization and inhibited translation. Based on this, we propose a model in which “structural processing” of *timP* mRNA enables tight inhibition by TimR in nonpermissive conditions, and TimP synthesis only upon TimR depletion.

## Significance

Transcription–translation coupling refers to when translation initiates during mRNA transcription. While beneficial at most genes, this phenomenon constrains tight gene repression. At many type I toxin genes, cotranscriptional translation is bypassed by a two-step mechanism. First, intramolecular structure renders the nascent mRNA translationally inactive, while subsequent ribonucleolytic processing generates an active mRNA. Second, the active mRNA is silenced by an antisense small RNA (sRNA). Contrary to this established mechanism, we here suggest an alternative mechanism for bypassing cotranscriptional translation. Instead of ribonucleolytic processing, the nascent *timP* mRNA is activated through a structural transition, which involves the formation of a pseudoknot. The active mRNA is specifically targeted by the sRNA TimR, which destabilizes the pseudoknot to inhibit translation.

In many bacterial species, mRNAs are often translated while transcription is ongoing, a phenomenon called transcription-translation coupling (1). While this mechanism may be beneficial for the expression of most genes, it becomes suboptimal for genes requiring tight repression at the mRNA level. Classical examples are type I toxin–antitoxin systems (T1TAs) in Gram-negative bacteria, where transcription-translation coupling is avoided through folding of the nascent mRNA (2, 3). The toxin gene of T1TAs is typically transcribed into a translationally inactive mRNA, in which intramolecular mRNA structure prohibits access for the 30S ribosomal subunit to the ribosome binding site (RBS) (4–7). To enable translation, the mRNA undergoes processing, upon which the toxin RBS (5, 6), the RBS of an upstream open reading frame (uORF) (8), or an upstream ribosome stand-by site (4, 9–13), becomes available for 30S binding. The antitoxin, a small antisense RNA, specifically targets the processed form of the mRNA to inhibit toxin translation (5, 9, 14–19). The long duplex formed between the toxin mRNA and the small antitoxin RNA is often subsequently cleaved by RNase III, resulting in a truncated and irreversibly translation-incompetent mRNA (4, 5, 8–10, 18). This two-step mechanism—production of a translationally inactive mRNA followed by processing and antitoxin-mediated inhibition—ensures tight regulation to avoid inadvertent toxin expression (3).

**Fig. 1.**
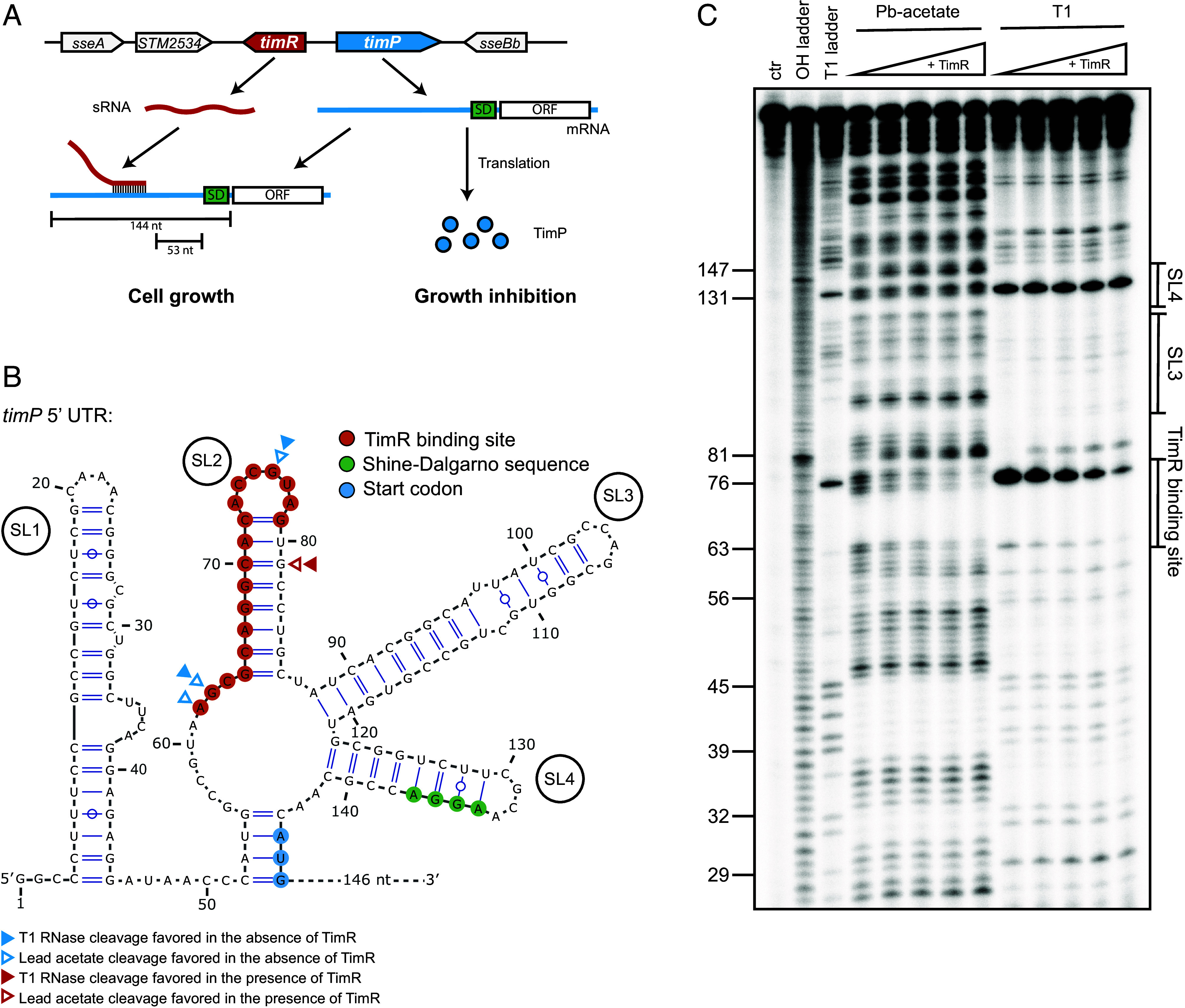
Secondary structure of *timP* 5′UTR indicates a noncanonical mechanism of sRNA-mediated regulation. (*A*) Schematic representation of the *timPR* T1TAs in *Salmonella enterica* subsp. enterica serovar Typhimurium. (*B*) Secondary structure representation of the *timP* 5′UTR based on structural conservation and structure probing. The TimR-binding site, SD sequence, and start codon is highlighted in red, green, and blue, respectively. Arrows show positions amenable to strong changes in lead(II)-acetate or RNase T1 cleavage upon TimR-binding. (*C*) Representative structure probing experiment of radioactive labeled *timP* mRNA with increasing concentrations of unlabeled TimR sRNA. Ctr: untreated RNA, OH ladder: denatured RNA subjected to alkaline hydrolysis, T1 ladder: denatured RNA subjected to RNase T1 cleavage.

We recently showed that the T1TAs *timPR* is conserved in enterobacteria (19). The *timP* mRNA, initially annotated as noncoding RNA RyfA (20), encodes a small toxic inner membrane protein that, upon overexpression, induces membrane leakage and cell growth inhibition (19). A puzzling peculiarity of the *timPR* system was that *timP* mRNA did not undergo ribonucleolytic processing (19), suggesting that overcoming inhibited transcription-translation coupling here relies on an alternative mechanism. TimR is a small RNA (sRNA) that inhibits TimP synthesis (Fig. 1*A*) by binding to a complementary region in the *timP* 5′UTR (19). Interestingly, the predicted TimR-binding site is located about fifty nucleotides upstream of the *timP* RBS. This indicates that TimR-dependent translation inhibition does not directly block 30S access to the *timP* RBS but rather works through a noncanonical mechanism.

In the current study, we investigated the mechanism of translation initiation at the *timP* mRNA, as well as mechanistic aspects of TimR-dependent inhibition. We propose that *timP* adopts mutually exclusive structural conformations, of which only one permits translation. The translation-competent conformation harbors a pseudoknot structure, which is essential for translation to occur. Our data suggest that TimR preferentially binds to the translation-competent conformation of *timP*, which destabilizes the pseudoknot, thereby providing a mechanism for TimR-dependent inhibition of translation. Based on this, we suggest an alternative two-step mechanism for controlling expression of a type I toxin. Instead of enzymatic processing, the primary *timP* mRNA undergoes a structural transition from an inactive to an active conformation. Next, TimR targets the active conformation of *timP*, thereby preventing toxin expression.

## Results

### A Conserved Secondary Structure Sequesters the *timP* RBS.

In our previous work, Northern blot analysis failed to detect enzymatic processing of *timP* mRNA in vivo, and in vitro translation showed that the primary transcript is efficiently translated and can be inhibited by TimR (19). Thus, the mechanistic basis for overcoming inhibited transcription–translation coupling at the *timP* mRNA must differ from the one established for many other T1TAs. To get more insight into the molecular basis for *timP* translation and TimR-binding, the secondary structure of the *timP* 5′UTR was analyzed. Alignment of homologous *timP* sequences indicated high structural conservation despite considerable sequence variation (*SI Appendix*, Fig. S1). The predicted structure consists of four stem-loops, of which stem-loop 2 (SL2) harbors the TimR-binding site, and SL4 sequesters the Shine-Dalgarno (SD) sequence (Fig. 1*B*). Next, we experimentally analyzed the secondary structure of the *timP* 5′UTR in vitro. To this end, radioactively labeled *timP* mRNA incubated with increasing concentrations of TimR was cleaved by lead(II)-acetate or RNase T1 (Fig. 1*C*). The probing results strongly support the predicted structure of *timP* (Fig. 1*B* and *SI Appendix*, Fig. S1*B*). In the presence of TimR, the loop of SL2 was protected from cleavage by both probes (e.g., G76), consistent with this region becoming double-stranded upon TimR-binding (Fig. 1*C*). By contrast, addition of TimR resulted in increased cleavage at the 3′ side of SL2 (e.g., G81) indicating that TimR-binding destabilizes SL2 (Fig. 1*C*). Outside SL2, TimR conferred minor changes in the cleavage pattern at positions 57 to 58, 142 to 144, and around position 165 (Fig. 1*C*). Probing also confirmed that the SD sequence is trapped in SL4. Strong lead and T1 cleavages were detected in the SL4 loop (e.g., G131), while the absence of cleavages in the stem was apparent not only in refolded *timP* but also in the denatured T1 ladder, indicating an unusually stable structure (Fig. 1*C*). Taken together, the *timP* 5′UTR adopts a secondary structure consisting of four stem-loops. TimR-binding results in local structural changes in SL2, and the SD sequence is trapped in SL4.

### Translation of *timP* mRNA Relies on Elements far Upstream of the RBS.

**Fig. 2.**
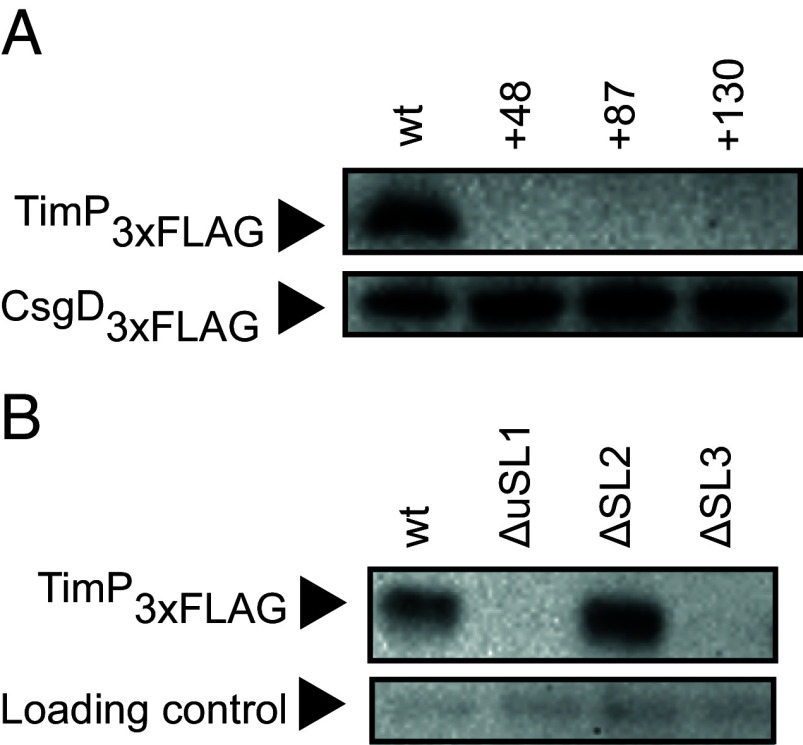
Stem-loops SL1 and SL3 in the *timP* mRNA are essential for translation. (*A*) In vitro translation of 5 nM of *timP-3xflag* mRNA and truncations thereof. The *csgD-3xflag* mRNA served as loading control. (*B*) In vitro translation of 5 nM of *timP-3xflag* and corresponding stem-loop deletion mutants. An unspecific band served as loading control.

Despite the SD sequence being occluded in SL4, *timP* mRNA is translated both in vivo and in a reconstituted in vitro translation system (Fig. 2 *A* and *B* and *SI Appendix*, Fig. S3 *A*, *C*, and *D*). Importantly, the SD sequence is required for translation, since deletion of SL4 abolishes translation (*SI Appendix*, Fig. S2 *A* and *B*). Occlusion of a SD sequence in a stem-loop is a recurring theme in T1TAs, and different mechanisms for overcoming this obstacle to translation initiation have been described. For instance, translation initiation at the structurally inaccessible SD of *hok* mRNA relies on translational coupling with the uORF *mok* (8). The *timP* 5′UTR harbors a putative start codon at positions 53 to 55 and an in-frame stop codon at position 77 to 79. This putative uORF overlaps with the TimR-binding site. To assess whether *timP* translation relies on the putative uORF, a point mutation that changes its start codon from AUG to AAG (mutation M1, *SI Appendix*, Fig. S2*C*) was introduced. However, this did not affect *timP* translation (*SI Appendix*, Fig. S2*B*), thereby ruling out translational coupling as a plausible mechanism for translation initiation at *timP*. By contrast, a mutation that destabilizes the lower part of SL1 (mutation M7) dramatically reduced translation in vitro, and restoration of SL1 by the compensatory mutation M7’ (*SI Appendix*, Fig. S2*C*) fully restored translation (*SI Appendix*, Fig. S2*B*). To further identify determinants required for translation, the mRNA was truncated from the 5′ end to give *timP*+48, *timP*+87, and *timP*+130 mRNAs (*SI Appendix*, Fig. S2*C*). None of the truncated mRNAs gave translation products in vitro or in vivo (Fig. 2*A* and *SI Appendix*, Fig. S3*A*). The translation deficiency of *timP*+130 was surprising, considering that in this variant formation of SL4 is abolished. However, this may be due to the formation of an alternative stem-loop structure sequestering the RBS (*SI Appendix*, Fig. S2*D*). By contrast to the in vitro translation system, in which RNAs are stable, the *timP*+48 and *timP*+130 mRNAs were undetectable in vivo (*SI Appendix*, Fig. S3*B*). Possibly, the strongly reduced mRNA levels in these mutants may reflect instability due to the loss of a protective stem-loop structure at the 5′ end (*SI Appendix*, Fig. S2*C*). However, the in vitro translation results, together with the M7 mutation shown in *SI Appendix*, Fig. S2, indicate that SL1 harbors a determinant important for translation initiation. Next, the upper part of SL1 (mutant uSL1), the complete SL2, or SL3, was deleted from *timP* mRNA (*SI Appendix*, Fig. S2*A*), respectively. Consistent with the importance of SL1 shown above, the uSL1 mutant was translationally inactive in vitro and in vivo (Fig. 2*B* and *SI Appendix*, Fig. S3*C*). Surprisingly, while deletion of SL2 did not affect translation, the SL3 deletion resulted in dramatically reduced translation rates (Fig. 2*B* and *SI Appendix*, Fig. S3*C*). Once again, the *timP* deletion variants presented lower mRNA stability than the wild-type in vivo (*SI Appendix*, Fig. S3*B*). Taken together, translation of *timP* mRNA does not rely on an uORF but requires elements in both SL1 and SL3.

### A Pseudoknot between SL1 and SL3 Is Essential for *timP* Translation.

**Fig. 3.**
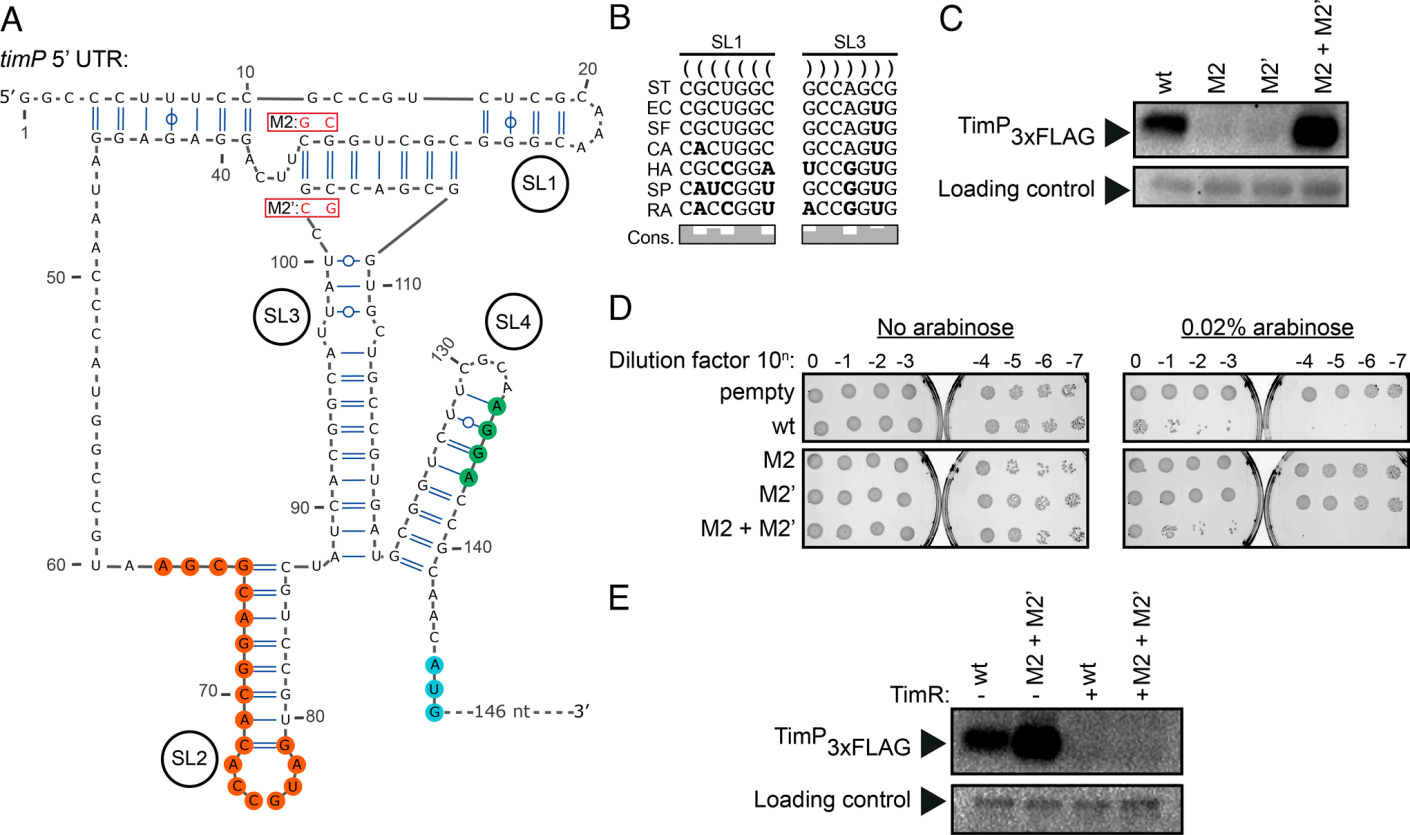
A pseudoknot structure is essential for translation of the *timP* mRNA. (*A*) Secondary structure representation of the *timP* 5′UTR. Mutations M2 and M2’ designed to disrupt the pseudoknot are highlighted in red. (*B*) Alignment of complementary regions of *timP* SL1 and SL3 in the indicated enterobacterial species, ST: *S. enterica* subsp. enterica serovar Typhimurium, EC: *Escherichia coli*, SF: *Shigella flexneri,* CA: *Citrobacter amalonaticus,* HA: *Hafnia alvei*, SP: *Serratia proteamaculans*, RA: *Rahnella aquatilis*. (*C*) In vitro translation of 5 nM of *timP-3xflag* with or without the indicated mutations. An unspecific band served as loading control. (*D*) Toxicity induced by overexpression of *timP* or the indicated pseudoknot mutants after 45 min induction with 0.02% arabinose. (*E*) In vitro translation assay of wild-type *timP* and the M2+M2’ mutant in the absence or presence of TimR.

Given the requirement of both SL1 and SL3 for *timP* translation, the possibility of an interaction between these two stem-loops, that is, formation of a pseudoknot structure, was investigated. Indeed, a putative seven base–pair interaction between uSL1 and SL3 was identified (Fig. 3*A*), supported by covariation of base-pairing among different enterobacterial *timP* homologs (Fig. 3*B*). To experimentally test whether *timP* translation requires formation of the predicted pseudoknot structure, two mutations were introduced. The *timP-*M2 and *timP*-M2’ mutants were designed to disrupt pseudoknot-formation on the SL1 or SL3 side, respectively (Fig. 3*A*), while combining the two mutations (*timP*-M2+M2’) should restore the interaction. To test whether SL3 forms a pseudoknot with SL1, as predicted, we designed a DNA oligo complementary to SL3 downstream of the SL1 interaction site. We reasoned that annealing of the oligo should be more efficient if the pseudoknot was disrupted by a mutation. The DNA oligo was annealed to *timP*, *timP-*M2’, or *timP*-M2+M2’ and the formation of a heteroduplex was assayed by RNase H cleavage. While the wild-type and M2+M2’ mutant only showed weak RNase H cleavage, the M2’ mutation resulted in a strong cleavage (*SI Appendix*, Fig. S3*E*). Hence, in *timP*-M2’, SL3 was accessible for oligo binding, indicating disrupted pseudoknot-formation. Strikingly, each of the single mutations completely abolished translation of *timP* mRNA in vitro, while the compensatory mutant mRNA gave translation levels even exceeding that of wild-type mRNA (Fig. 3*C*). Hence, the formation of a pseudoknot between SL1 and SL3 is essential for *timP* translation. Similar to the in vitro results, the M2 and M2’ mutations abolished translation in vivo, and could be rescued by restoring pseudoknot base-pairing (*SI Appendix*, Fig. S3*D*). Northern blot analysis showed that mutations that disrupt the pseudoknot (M2 and M2’) negatively affect mRNA levels, indicating that the pseudoknot promotes mRNA stability (*SI Appendix*, Fig. S3*B*). The effect of the pseudoknot on the translation of *timP* in vivo was further tested by a toxicity assay. Serial dilutions of cultures expressing *timP*, *timP-*M2, *timP-*M2’, or *timP*-M2+M2’ were spotted onto agar plates. Uninduced cultures served as controls. While expression of wild-type *timP* resulted in a toxic phenotype, as expected (19), induction of *timP-*M2 or *timP-*M2’ allowed growth to the same extent as the strain harboring an empty vector (Fig. 3*D*). Importantly, the compensatory mutant phenocopied the toxic effect of the wild-type allele (Fig. 3*D*). TimR fully inhibited translation not only of wild-type *timP* but also the *timP*-M2+M2’ mutant in vitro (Fig. 3*E*). We conclude that translation initiation at the *timP* mRNA requires the formation of a pseudoknot structure between SL1 and SL3.

### TimR Binds the Pseudoknot-Containing Conformation of *timP* mRNA.

**Fig. 4.**
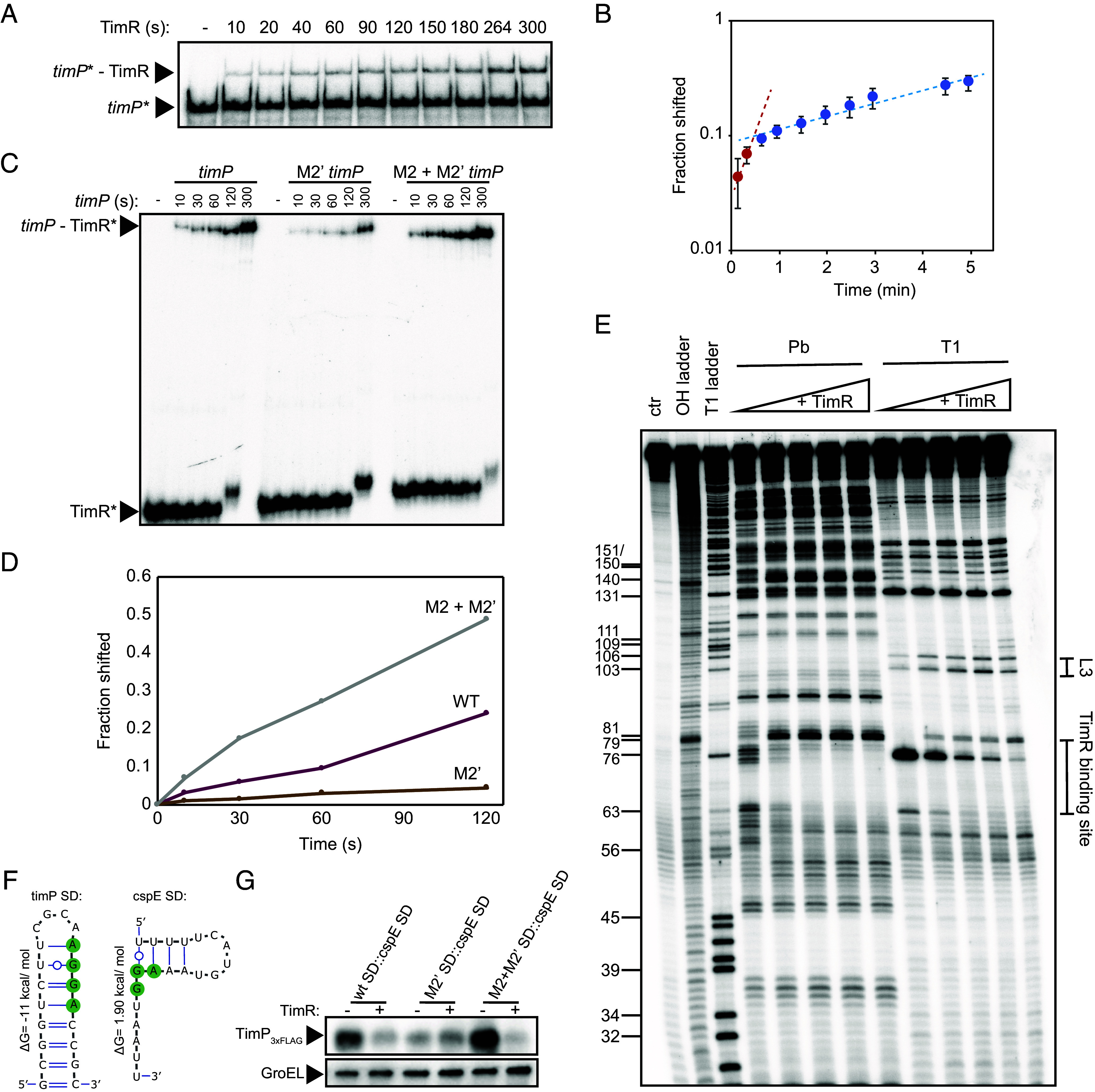
TimR-binding destabilizes pseudoknot-formation at *timP* mRNA. (*A*) Time-course electromobility shift assay of radioactively labeled *timP* mRNA in the presence of unlabeled TimR sRNA. (*B*) Quantification of three independent experiments as shown in panel *A*. Average values and SD are shown. The two different rates are plotted as trendlines. (*C*) Time-course electromobility shift assay of radioactively labeled TimR in the presence of unlabeled wild-type, M2’ or M2+M2’ *timP* mRNA. (*D*) Quantification of the gel shown in panel *C*. (*E*) Structure probing of radioactive labeled *timP* M2+M2’ mRNA in the presence of increasing concentrations of unlabeled TimR. Ctr: untreated RNA, OH ladder: denatured RNA subjected to alkaline hydrolysis, T1 ladder: denatured RNA subjected to RNase T1 cleavage. (*F*) Secondary structure representation of the *timP* SL4 and the equivalent but unstructured region from the *cspE* mRNA. (*G*) Western blot monitoring in vivo expression of *timP-3xflag* with replacement of SL4 by *cspE* SD sequence. *timP-3xflag* was expressed from a plasmid for 45 min in the presence of 0.02% arabinose. GroEL was used as a loading control.

In T1TAs, the antitoxin sRNA specifically targets the processed and translationally active mRNA. The *timP* mRNA differs in that it does not undergo enzymatic processing (19) but instead relies on a specific structural fold including a pseudoknot to allow translation (Fig. 3). Based on this, we hypothesized that *timP* requires a structural transition from a translation-incompetent to a translation-competent conformation, only the latter one containing the pseudoknot. In analogy with other T1TAs, TimR should then specifically target the pseudoknot-containing mRNA. To test this, we first monitored the binding between labeled *timP* and unlabeled TimR as a function of time using electromobility shift assays (Fig. 4*A*). Interestingly, quantification indicated two different binding rates, an initial fast rate followed by a much slower rate (Fig. 4*B*). Since only a fraction of the *timP* molecules bound to TimR at the fast rate, the pool of *timP* molecules may consist of different structural conformations, of which only a minority is binding-competent at early time points. By contrast, the slow rate of binding at later time points might reflect a slow transition rate from a binding-incompetent to the binding-competent conformation. Notably, although only a small fraction of *timP* mRNA was bound by TimR throughout the binding assay (*SI Appendix*, Fig. S4*A*), TimR completely abolished *timP* translation during in vitro translation (Fig. 3*E*), despite similar concentrations of *timP* and TimR in both assays (*Materials and Methods*). This suggests that *timP* adopts different conformations and that the same conformation that promotes translation is competent for TimR-binding. To test this, we performed time-course binding assays with labeled TimR and an excess of unlabeled *timP*, *timP*-M2’, or *timP*-M2+M2’ RNAs. Interestingly, while the M2’ mutation strongly reduced the rate of complex formation compared to wild-type *timP* (Fig. 4 *C* and *D*), the M2+M2’ mutant not only restored but increased the binding rate beyond wild-type levels (Fig. 4 *C* and *D*). Thus, the M2+M2’ mutant mRNA both binds TimR faster (Fig. 4*D*) and is more efficiently translated in the absence of TimR (Fig. 3*C*). This indicates that TimR preferentially binds to the pseudoknot-containing and translationally active conformation of *timP* mRNA.

### TimR-Binding Disrupts the Pseudoknot in timP mRNA.

Since TimR inhibits *timP* translation (Fig. 3*E*), and translation itself requires pseudoknot-formation (Fig. 3*C*), we asked whether TimR-binding disrupts the pseudoknot. The *timP*-M2+M2’ mRNA was subjected to structure probing in the presence or absence of TimR (Fig. 4*E*). For this, we used the compensatory mutant mRNA instead of wild-type *timP* since a greater fraction appears to form the pseudoknot (Figs. 3*C* and 4*D*). This should facilitate detection of structural changes in the pseudoknot against the background of other conformations lacking the pseudoknot. Introduction of the M2+M2’ mutation did not affect the overall structure compared to wild-type *timP* (Figs. 1*C* and 4*E*), and addition of TimR resulted in the same local changes in cleavage pattern as previously seen with wild-type *timP* (Fig. 1*C*). However, the presence of TimR also resulted in increased cleavages at positions G103 and G106 within the SL3 part of the pseudoknot (Fig. 4*E*), indicating destabilization of the pseudoknot upon TimR-binding. We also observed slightly increased cleavage at positions G109 and G111 in SL3 (Fig. 4*E*). Since the pseudoknot-disrupting mutations (M2 and M2’) completely abolished translation of *timP* (Fig. 3*C*), it was difficult to test the effect of TimR on these mutants. In an attempt to bypass the pseudoknot-dependent translation at *timP* mRNA, we replaced SL4, which harbors the SD sequence, with the equivalent but unstructured region of the *cspE* mRNA (Fig. 4*F*). Analyzing TimP expression from this variant in vivo gave several interesting results (Fig. 4*G* and *SI Appendix*, Fig. S4*B*). First, TimP expression was significantly higher in wild-type and M2+M2’ *timP* compared to the M2’ mutant. Second, the M2’ mutation conferred an intermediate expression level. Third, coexpression of TimR reduced TimP expression when the pseudoknot was intact (wild-type and M2+M2’ mutant) but had no effect on expression from the M2’ mutant. This indicates that the pseudoknot enhances translation initiation rates not only if the SD sequence is sequestered in a stable stem-loop but also in the presence of an unstructured and accessible SD sequence. Moreover, TimR-mediated inhibition of translation is strictly dependent on the pseudoknot. In conclusion, *timP* mRNA adopts different conformations, one of which carries a pseudoknot between SL1 and SL3. The latter conformation is translationally active and confers binding to TimR. Binding of TimR disrupts the pseudoknot, and thereby, inhibits translation initiation.

## Discussion

The activities of type I toxins have detrimental effects on cell physiology, but may be beneficial for the genetic elements in which toxin genes reside. The plasmid-encoded Hok toxin is produced only when the genes encoding both toxin and antitoxin are absent from the cell, that is, in plasmid-free cells. This entails killing of plasmid-free cells and stable maintenance of the plasmid in the population (8, 21). The TisB and DinQ toxins are produced upon DNA damage, a condition that seriously threatens propagation of the genome that harbors their genes (9, 22, 23). In principle, T1TAs may also protect bacterial populations from propagation of lytic phages (24). Hence, the expression of toxins only occurs under very specific conditions and therefore requires unusually tight regulation.

While some type I toxins are controlled at the level of transcription, all are tightly regulated at the posttranscriptional level. In Gram-negative bacteria, transcription of these genes generates primary transcripts that are translationally inert and require processing to become translationally active. For instance, ribonucleolytic cleavage generates a 5′ truncated *tisB* mRNA, which renders a ribosome stand-by site accessible for 30S binding, and thus permits translation initiation. At the same time, the exposed stand-by site is the target of the antitoxin IstR-1 (4, 9, 12, 13). This two-level regulation prohibits translation of the toxin both during transcription and after processing, as long as the antitoxin is present.

The *timPR* system presents a different solution to the same problem, since *timP* mRNA does not undergo enzymatic processing (19). The primary transcript is efficiently translated in vitro (Figs. 2 and 3), while truncations of the mRNA from the 5′ end result in loss of translation (Fig. 2*A*), rather than activation. This strongly indicates that the primary *timP* transcript does not require processing to become translationally active and suggests an alternative mechanism to overcome the inhibition of cotranscriptional translation.

The solution to this may be reflected in some of the seemingly paradoxical results obtained from the biochemical analyses presented in this paper. In the binding experiments shown in *SI Appendix*, Fig. S4*A*, where TimR was provided in large excess over radiolabeled *timP*, only a minor fraction of *timP* formed a complex with TimR (*SI Appendix*, Fig. S4*A*). However, under comparable conditions, TimR completely inhibited *timP* translation (Fig. 3*E*). One plausible explanation to this dichotomy is that *timP* can adopt different structural conformations, only one of which is competent for translation initiation as well as TimR-binding. For clarity, we will henceforth denote the TimR-binding- and translation-*incompetent* conformation(s) *timP_I_* and the binding- and translation-*competent* conformation *timP_A_*.

Time-course binding experiments revealed that a small fraction of *timP* molecules rapidly (association rate constant ~10^5^ M^−1^s^−1^) formed a duplex with TimR after mixing the two RNAs (Fig. 4*B*). After this initial fast binding, *timP*–TimR complexes continued to form, but at a much slower rate. The initial fast binding rate should reflect binding between TimR and a pre-existing pool of *timP_A_*, while the slow rate likely is determined by the rate by which *timP* molecules transition from *timP_I_* to *timP_A_*. Since *timP*–TimR duplex formation is nonreversible in the binding assays (*SI Appendix*, Fig. S4*C*), each binding event should disturb the equilibrium between *timP_I_* and *timP_A_* in the pool of nonbound *timP*. Re-establishing the equilibrium should involve further structural transitions from *timP_I_* to a *timP_A_*. From this follows that the complete TimR-dependent inhibition of translation observed in in vitro translation experiments (Fig. 3*E*) reflects that each transition from *timP_I_* to *timP_A_* is followed by rapid TimR-binding to *timP_A_*, thereby prohibiting translation initiation to occur.

Based on this, we propose a model for the expression of TimP in vivo (*SI Appendix*, Fig. S5). According to this, the nascent *timP* mRNA initially folds into conformation *timP_I_*. In conditions where TimP expression is unfavorable, TimR will be in excess of *timP* mRNA, so that mRNAs transitioning from *timP_I_* to *timP_A_* are bound and inactivated by TimR. By contrast, in conditions where TimP expression is favorable, the ratio between *timP* and TimR may change, either by repression of TimR transcription, activation of *timP* transcription, or both, enabling translation of more *timP* mRNA molecules that transition from *timP_I_* to *timP_A_* to escape inhibition. In addition, environmental changes, such as fluctuations in the Mg^2+^ concentration or the presence of a small ligand, could potentially influence the structural transition, favoring the *timP_A_* conformation.

Based on the results presented here, the transition from *timP_I_* to *timP_A_* involves the formation of a pseudoknot in the *timP* 5′UTR. Structure probing experiments and conservation analysis showed that the *timP* SD sequence is trapped in the stable stem-loop structure SL4 (Fig. 1*B*). The fact that SL4 has a greater thermodynamic stability than the SD-sequestering stem-loop of the MS2 coat protein mRNA (−11 vs. −9 kcal mol^−1^) should prohibit 30S to access the SD from solution at any biologically meaningful time-scale (25, 26). Still, *timP* is efficiently translated both in vitro (Figs. 2 and 3) and in vivo (*SI Appendix*, Fig. S3). Introduction of truncations, deletions, and point mutations in the *timP* 5′UTR showed that a pseudoknot structure between SL1 and SL3 is essential for translation initiation (Fig. 3). Destabilizing the pseudoknot by a point mutation (M2’) not only abolished translation but also strongly decreased the association rate of TimR-binding. Thus, conformation *timP_A_* contains the pseudoknot while *timP_I_* does not. Interestingly, introduction of a compensatory mutation (M2+M2’) to restore the pseudoknot structure increased both translation and TimR-binding beyond that observed with wild-type *timP* (Fig. 3). This suggests that the *timP*-M2+M2’ mutant is more prone to form the pseudoknot than wild-type *timP*. We speculate that in the wild-type mRNA, there are at least two mutually exclusive folds of SL1: the internal base-pairing shown in Fig. 1*B*, and the pseudoknot (Fig. 3*A*). Due to mutation M2 weakening the base-pairing internal to SL1, the M2+M2’ mutations favor pseudoknot-formation. In other words, the *timP_A_* to *timP_I_* ratio is greater for *timP*-M2+M2’ than for wild-type *timP*. The increased ratio of pseudoknot-containing molecules in the M2+M2’ mutant also allowed detection of pseudoknot destabilization upon TimR-binding (Fig. 4*E*). Most likely, TimR-binding destabilizes the pseudoknot also in the wild-type mRNA, but since only a small fraction contains the pseudoknot, it becomes difficult to detect in experiments with bulk average readout. It will be interesting to see whether RNA structural processing is implicated in regulation of additional T1TAs. Interesting candidates may include some T1TAs in Gram-positive bacteria, including the *par* locus in *Enterococcus faecalis* (27) and *txpA/ RatA* in *Bacillus subtilis* (28), where the toxin mRNAs seem to be devoid of enzymatic processing.

Although the formation of the pseudoknot is essential for *timP* translation, the specific role for the pseudoknot in translation initiation remains unclear. In the case of *tisB*, translation initiation requires the recognition of a pseudoknot structure by ribosomal protein S1 and/or 30S (13). Cross-linking, immunoprecipitation, and deep sequencing experiments further suggested that recognition of the pseudoknot is followed by 30S/S1 movement along the *tisB* mRNA to reach the structured RBS (12). The fact that *timP* can be efficiently translated in a defined in vitro system with purified components speaks for recognition of the pseudoknot by components of the translation machinery also here. Hence, a plausible explanation is that S1 recognizes the pseudoknot structure, thereby recruiting 30S. The helicase activity of S1 could facilitate destabilization of SL4, followed by binding of the preinitiation complex to the RBS. Further unresolved issues include why TimR preferentially binds the pseudoknot-containing conformation of *timP*, and how binding of TimR destabilizes the pseudoknot. Answering these questions may require determination of the different structural conformations of *timP* at high resolution, for instance by cryogenic electron microscopy and single-molecule analysis.

Taken together, we show that a pseudoknot structure in a highly structured 5′UTR is an essential regulatory element for translation initiation, and provide evidence that structural rearrangement could act as an alternative “processing” step to obtain tight regulation of type I toxins. Finally, we suggest a mechanism of translation inhibition, wherein a base-pairing sRNA prevents pseudoknot-formation.

## Materials and Methods

### Bacterial Strains and Growth Conditions.

In this study, *S. enterica* subsp. *enterica* serovar Typhimurium strain SL1344 was used as the wild-type strain in all experiments. *Escherichia coli* strain TOP10 was used for cloning purposes. Bacteria were grown aerobically at 37 °C, 200 rpm in LB medium or M9 medium supplemented with chloramphenicol (12.5 µg/mL), unless otherwise specified. M9 minimal medium was further supplemented with 0.1% casamino acids, 0.4% glycerol, and 10 µg/mL thiamine. Expression from the P*_araBAD_* promoter was induced by addition of 0.02% or 0.2% L-arabinose at an OD_600_ of 0.3 for 45 or 30 min, respectively.

### Cloning and Strain Construction.

All plasmids and oligonucleotides constructed and/or used in this study are listed in *SI Appendix*, Tables S1 and S2, respectively. Plasmids encoding *timP* mutants were constructed by PCR using pYMB025 or pYMB023 as template, followed by DpnI treatment and ligation.

### In Vitro Transcription, Purification, Dephosphorylation, and Labeling of RNA.

RNA molecules were generated by in vitro transcription using the MEGAscript kit (LifeTechnologies). DNA templates carrying a T7 promoter were generated by PCR (*timP*-3xflag variants) or by oligonucleotide annealing followed by a Klenow fragment fill-in reaction (TimR variants). Transcription reactions were separated by denaturing polyacrylamide/8 M Urea gel electrophoresis, followed by gel extraction in elution buffer (10 mM magnesium acetate tetrahydrate, 500 mM ammonium acetate, 1 mM EDTA, 0.1% SDS), phenol-chloroform extraction, ethanol precipitation, and resuspension in sterile water. If appropriate, the in vitro transcribed RNAs were dephosphorylated, purified by phenol-chloroform extraction and ethanol precipitation, and radioactively labeled at the 5′-end using γ-^32^P-ATP and T4 PNK (ThermoFisher).

### Electromobility Shift Assays.

Electromobility Shift Assays were performed in 1× EMSA buffer (25 mM Tris-HCl pH 7.4, 100 mM NaCl, 1 mM MgCl_2_). RNA was denatured for 1 min at 95 °C in sterile water, cooled on ice for 2 min, diluted in EMSA buffer, and renatured for 5 min at 37 °C. For the rate experiments, 0.5 nM of labeled RNA was mixed with 500 nM unlabeled RNA, and samples were taken in time intervals. Samples were immediately separated in running native 6% polyacrylamide gels in 0.5% TBE buffer, at 200 V and 4 °C.

### RNA Structure Probing.

Labeled *timP* mRNA at a final concentration of 25 nM was mixed with increasing concentrations of cold TimR sRNA, heated at 95 °C, cooled on ice, and renatured in 1× TMN buffer (20 mM Tris-HCl pH = 7.4, 100 mM NaCl, 2 mM MgCl_2_) at RT for 30 min. The RNA mix was supplemented with 10 µg yeast RNA, and 20 mM PbAc for 1 min, or 0.05 U RNase T1 for 30 s. The reactions were stopped by adding 5 µL 0.1 M EDTA and kept on ice, followed by ethanol precipitation. The samples were then diluted in sterile water and Gel loading buffer II (Invitrogen), heated at 95 °C, and separated on 8% polyacrylamide sequencing gels in 1× TBE buffer at 38 W. Single nucleotide ladders were prepared by subjecting denatured RNA to alkaline hydrolysis according to the manufacturer (Ambion). Single G-nucleotide ladders were prepared by RNase T1 cleavage of denatured RNA.

### Sequence and Structural Conservation Alignments.

TimR and *timP* sequences of *Serratia proteamaculans* 568, *Rahnella aquatilis* CIP 78.65 (sequence ID: CP003244.1), *Hafnia alvei* FB1 (sequence ID: CP009706.1), *E. coli* strain K-12 subst. MG1655 (sequence ID: CP097884.1), *E. coli* O157:H7 strain PNUSAE147325 (Sequence ID: CP136755.1), *Yersinia enterocolitica* strain 8081 (sequence ID: CP009846.1), *S. enterica* subsp. enterica serovar Typhimurium SL1344 (Sequence ID: FQ312003.1), *Citrobacter freundii* ATCC 8090 (Sequence ID: CP049015.1), *Citrobacter amalonaticus* Y19 (Sequence ID: CP011132.1), and *Shigella flexneri* 2002017 (Sequence ID: CP001383.1) were aligned using LocARNA (29).

### In Vitro Translation.

In vitro translation was carried out using PURExpress (New England BioLabs). The RNAs were denatured at 95 °C and cooled on ice, followed by renaturation in 1× TMN buffer at 37 °C for 20 min. For each reaction, 2 μL of component A, 1.5 μL of component B, and 1.5 μL RNA (5 nM *timP*, 500 nM TimR) were incubated at 37 °C for 20 min. Reactions were stopped with addition of 2× Glycine-SDS-PAGE sample buffer and analyzed by Western blotting.

### Western Blotting.

Bacterial cultures were pelleted, resuspended in lysis buffer (5 mM EDTA, 0.2% sodium lauroylsarcosinate, 1× PBS), lysed using FastPrep, and resuspended in Glycine-SDS-PAGE sample buffer. The samples were denatured at 95 °C for 5 min, separated on Mini-PROTEAN® TGX Stain-Free protein gels (Bio-Rad), and transferred to 0.2 μm pore size polyvinylidene difluoride membranes using the TransBlot TURBO transfer system (Bio-Rad). The membranes were blocked overnight in 5% bovine serum albumin in TBS supplemented with 0.05% TWEEN-20. FLAG-tagged proteins were detected with a 1:10,000 diluted HRP-conjugated anti-FLAG mouse antibody (Sigma-Aldrich), and GroEL was detected by a 1:50,000 diluted HRP-conjugated anti-GroEL mouse antibody (Sigma-Aldrich).

### Toxicity Assays.

Ten-fold dilution series of bacterial cultures were prepared in 0.9% NaCl. From each dilution, 2 µL was spotted on LA plates supplemented with chloramphenicol (12.5 µg/mL) and incubated overnight at 37 °C.

## Supplementary Material

Appendix 01 (PDF)

## Data Availability

All study data are included in the article and/or *SI Appendix*.
