# Supplementary material for: An RNA pseudoknot mediates toxin translation and antitoxin inhibition
Source: Proc Natl Acad Sci U S A. 2024 Jun 27;121(27):e2403063121. doi: 10.1073/pnas.2403063121 (PMC11228461; doi:10.1073/pnas.2403063121)
Supplement: Supplementary file 1 — Appendix 01 (PDF) [file pnas.2403063121.sapp.pdf]

Supporting Information for

An RNA pseudoknot mediates toxin translation and antitoxin inhibition

Athina Eleftheraki and Erik Holmqvist

Corresponding author: Erik Holmqvist
Email: erik.holmqvist@icm.uu.se

This PDF file includes:

Supporting text
Figures S1 to S5
Tables S1 to S2
SI References

Supplementary Materials and Methods

RNA extraction

Bacterial cultures were mixed with 0.2 volumes of stop solution (95% ethanol, 5% phenol) and immediately frozen in liquid nitrogen, after 30 min induction with 0.2% L-arabinose. Following centrifugation, bacterial pellet was resuspended in TE buffer (10mM Tris pH 8.0, 1mM EDTA), supplemented with 0.5 mg/ ml. 1% SDS was then added in the suspension and the samples were incubated at 64°C for 2 min. 0.1 volume of sodium acetate pH 5.2 and 1 volume of acidic phenol were added in the suspension and the samples were incubated for 6 min at 64°C, followed by centrifugation, chlorophorm extraction and ethanol precipitation. The extraxted RNA was resuspended in sterile water and stored at -20oC. The RNA integrity was determined by agarose gel electrophoresis.

Northern blot

Total RNA samples (3.5 μg) were loaded on a 6% denaturing polyacrylamide/ 8 M Urea gel electrophoresis. Radioactively labeled puC19 DNA/ MspI (HpaII) Marker (ThermoFischer) was used as a size ladder. The total RNA was transferred to a Hybond-N+ nitrocellulose membrane (Amersham, Cytiva), 0.36 A, 17 V at 4°C for 2 hours, followed by crosslinking by UV light exposure at 1200 mJ and prehybridization with church buffer (0.5 M sodium phosphate buffer pH 7.2, 1 M EDTA, 7% SDS) for 45 min at 42°C. Radioactively labeled EHO-690 (targeting 5S rRNA) or EHO-1344 (targeting *timP* mRNA) was added at the hybridization buffer and incubated overnight at 42°C. The membrane was then washed in 2x SSC/ 0.1% SDS, dried and exposed to a phosphor screen.

RNase H cleavage assay

Renatured *in vitro* transcribed and radioactively labeled *timP* mRNA (47 nM) was incubated for 10 min with EHO-2502 (6.25 μM) at 37°C, in 1x TMN buffer. 5 U RNase H (New England Biolabs) or sterile water and RNase H buffer (New England Biolabs) were added, and incubated for 12 min at 37°C. Following addition of 25 μM EDTA, the samples were phenol-chloroform extracted and precipitated with ethanol. Redissolved RNA was denatured at 95°C for 2 min and loaded on a 6% denaturing gel at 25 V. Radioactively labeled puC19 DNA/ MspI (HpaII) Marker (ThermoFischer) was used as a size ladder. Subsequently the gel was exposed to a phosphor screen overnight.

Electromobility Shift Assays

Electromobility Shift Assays were performed in 1x EMSA buffer (25 mM Tris-HCl pH 7.4, 100 mM NaCl, 1 mM MgCl2). RNA was denatured for 1 min at 95ºC in sterile water, cooled on ice for 2 min, diluted in EMSA buffer, and renatured for 5 min at 37ºC. Labeled RNA at a final concentration of 0.5 nM was mixed with 500 nM of unlabeled RNA for 20 min at 37ºC. For the time-course experiments, 0.5 nM of labeled RNA was mixed with 500 nM unlabeled RNA for 10 min in 1x EMSA buffer. Renatured RNA at 500 nM was added in the reaction mix and samples were taken in time intervals. Samples were immediately separated in running native 6% polyacrylamide gels in 0.5% TBE buffer, at 200V and 4°C.

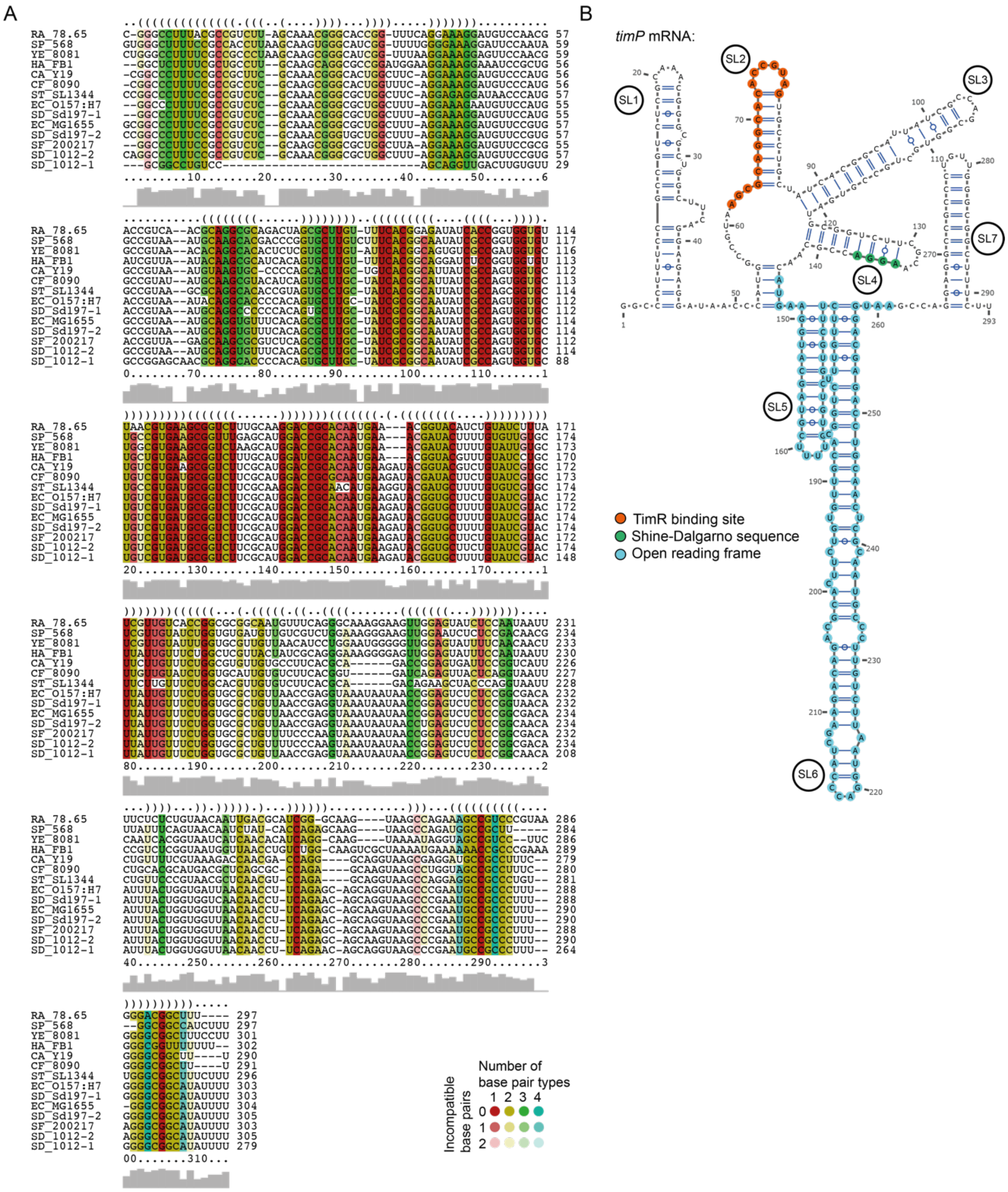

Fig. S1. The *timP* 5'UTR harbors a conserved secondary structure. (A) Alignment of the *timP* sequences from different enterobacterial species using LocARNA, ST: *Salmonella enterica* subsp. enterica serovar Typhimurium, EC: *Escherichia coli*, SF:*Shigella flexneri,* CA: *Citrobacter amalonaticus,* HA: *Hafnia alvei*, SP: *Serratia proteamaculans*, RA: *Rahnella aquatilis,* YE: *Yersinia enterocolitica,* CF: *Citrobacter freundii*. (B) Model of *timP* mRNA based on secondary structure data, conservation alignment, and structure prediction data, as presented also in Andresen L *et al.* (1)

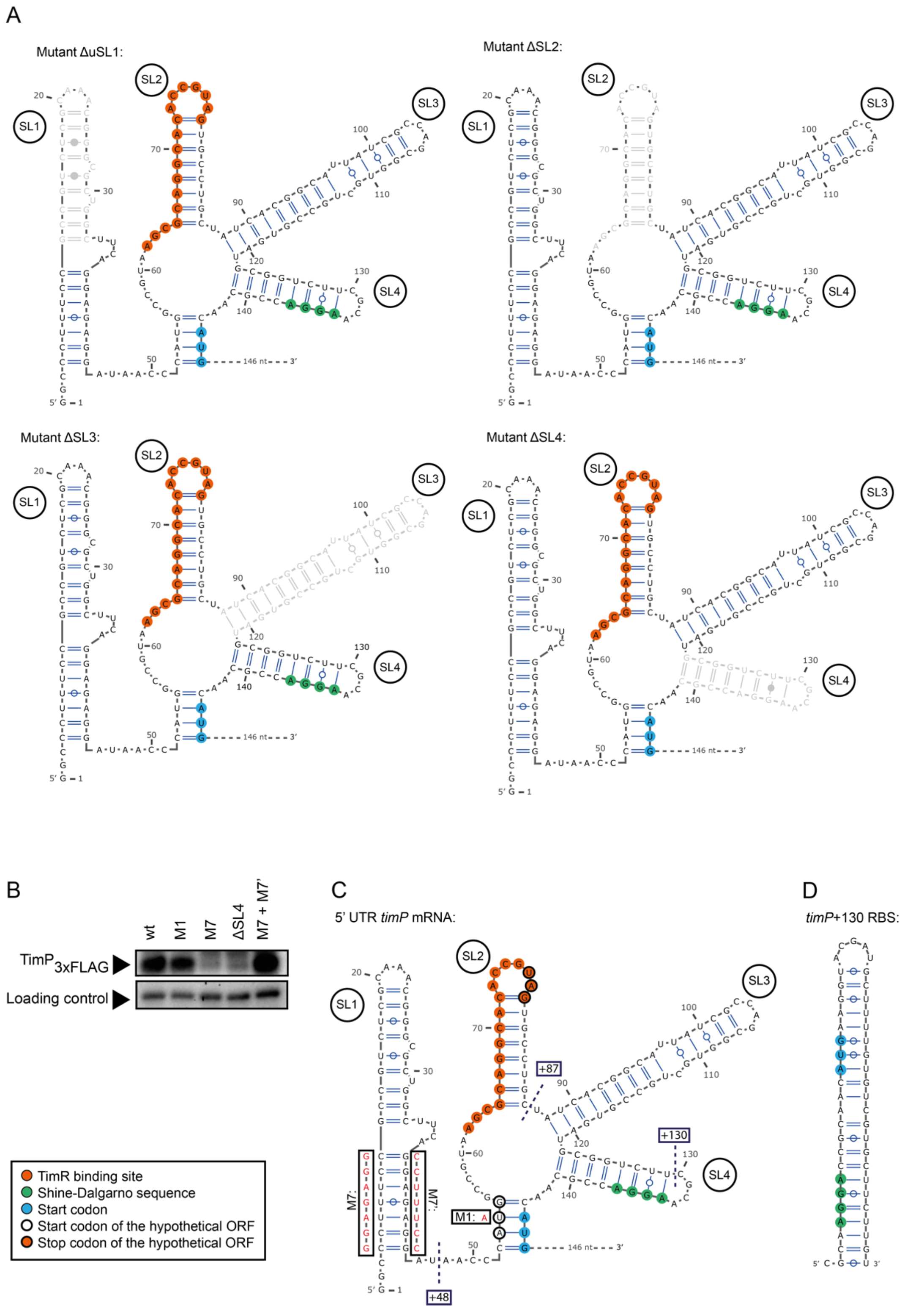

Fig. S2. (A) Secondary structure of the *timP* 5'UTR with indicated deletions (light grey). (B) *In vitro* translation of *timP-3xflag* mRNA and the indicated mutants thereof. An unspecific band served as a loading control. (C) Secondary structure of the *timP* 5'UTR with indicated mutations and truncation points. (D) Predicted structure at the RBS of *timP*+130.

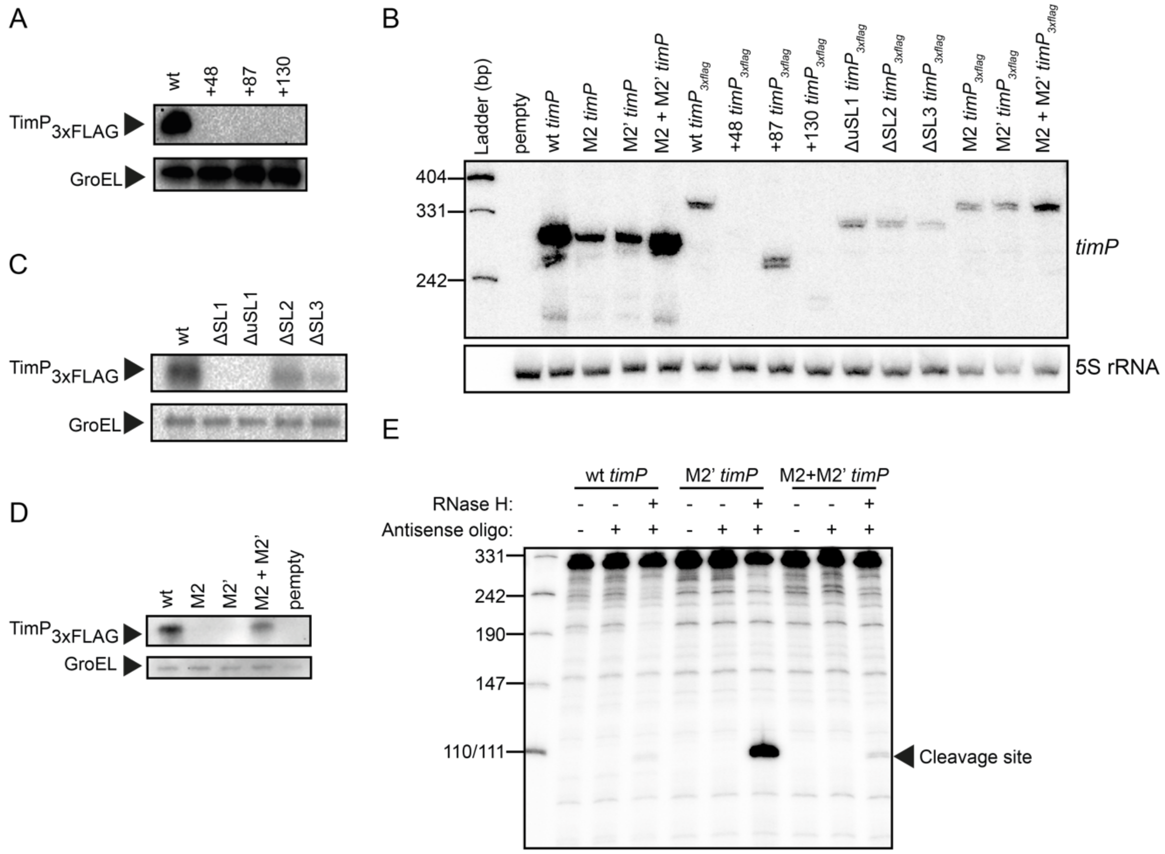

Fig. S3. (A) Western blot monitoring *in vivo* expression of *timP-3xflag* with 5’ truncations. The truncations are indicated in Fig. S2, *timP-3xflag* was expressed from a plasmid for 45 min in the presence of 0.02% arabinose. GroEL served as a loading control. (B) Northern blot analysis of *timP* mRNA and mutants thereof expressed from an arabinose-inducible promoter on a plasmid. Probing of 5S rRNA was used as loading control. (C-D) Western blot monitoring *in vivo* expression of *timP-3xflag* stem-loop deletion (C) or point mutations (D). The mutations are indicated in Fig. S2. GroEL served as a loading control. (E) Formation of a heteroduplex between an antisense oligo and the SL3 loop was tested by RNase H cleavage for wt *timP*, *timP*-M2’ or *timP*-M2+M2’.

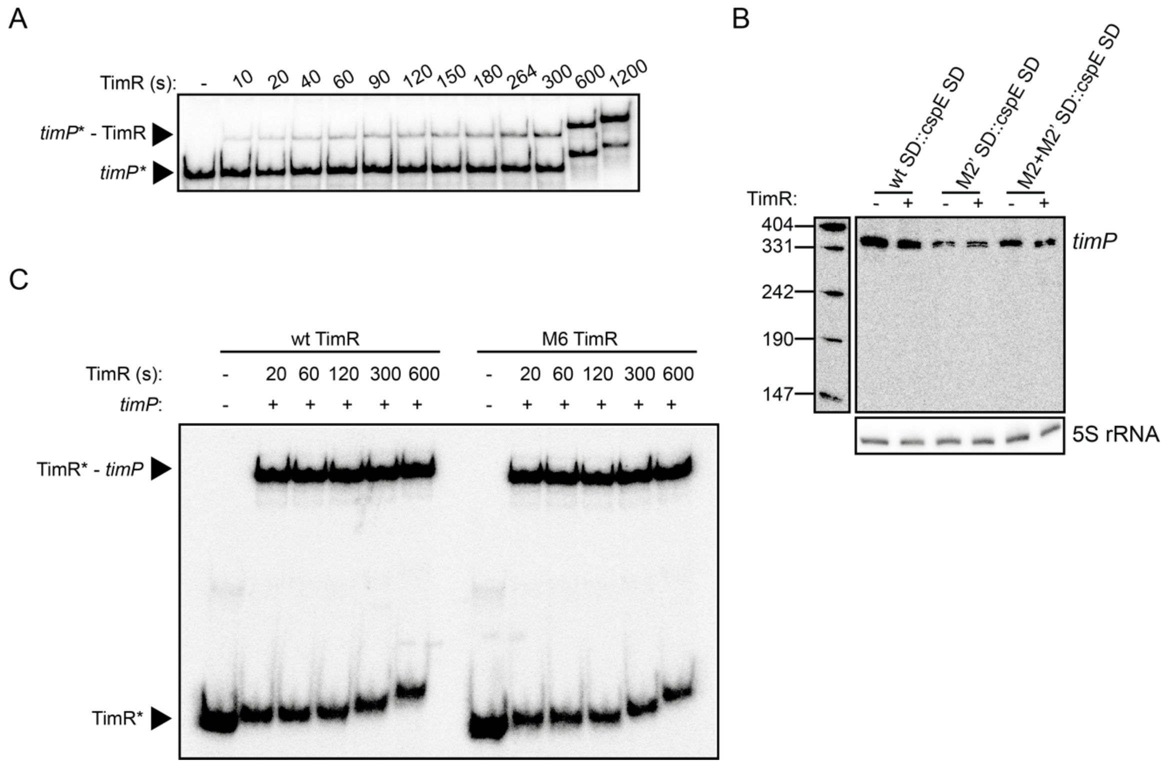

Fig. S4. (A) The same Electromobility Shift Assay as shown in Figure 4A including extended time points. Radioactive labeled TimR was incubated with an excess of unlabeled *timP* mRNA. (B) Northern blot analysis of the *timP* SD::*cspE* SD mRNA, and mutants thereof, expressed from an arabinose-inducible promoter on a plasmid. Probing of 5S rRNA was used as loading control. (C) Electromobility Shift Assay where radioactively labeled TimR pre-bound to unlabeled *timP* was challenged with an excess of unlabeled TimR or a binding-incompetent mutant (M6 TimR) after which samples were collected in time intervals.

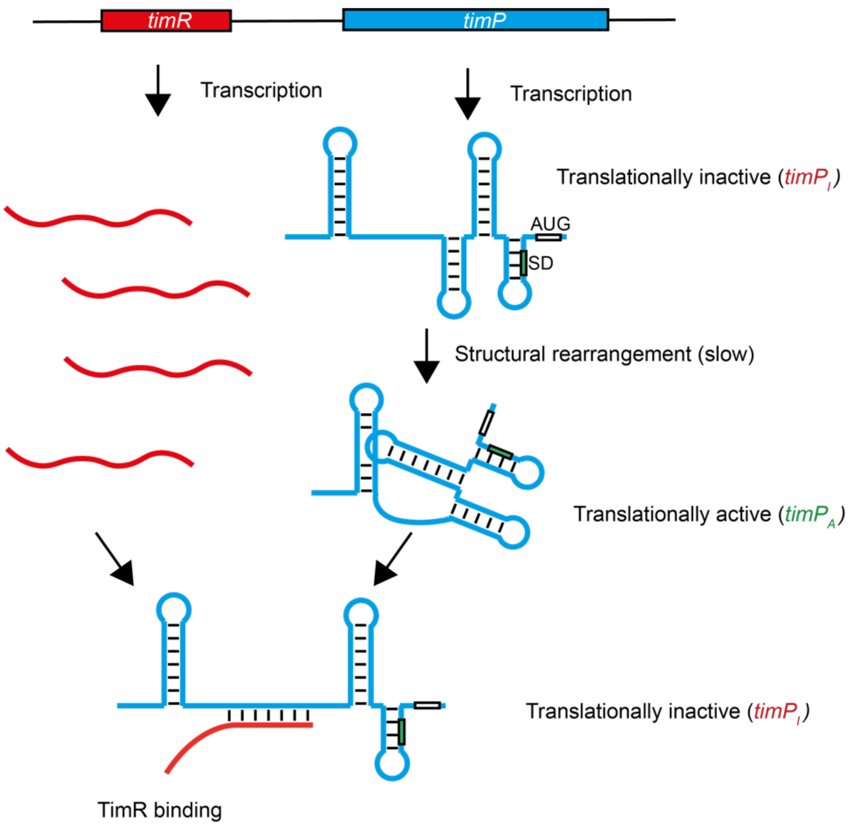

Fig. S5. Model for post-transcriptional regulation of the *timPR*. Transcription produces a translationally inactive *timP* mRNA, which subsequently becomes translationally active through a structural transition involving formation of a pseudoknot. TimR targets the translationally active structure, which destabilizes the pseudoknot and inhibits *timP* translation.

Table S1. Plasmids used in the study.

Plasmid	Description/ Source	Selection marker	Template
pBAD33	Empty vector (2)	CmR	
pYMB023	*timP* (1)	CmR	
pYMB025	*timP-3x flag* (1)	CmR	
pAE003	M7 *timP-3x flag*	CmR	pYMB025
pAE004	ΔSL4 *timP-3x flag*	CmR	pYMB025
pAE005	M7+M7' *timP-3x flag*	CmR	pYMB025
pAE006	M1 *timP-3x flag*	CmR	pYMB025
pAE007	+48 *timP-3x flag*	CmR	pYMB025
pAE008	+87 *timP-3x flag*	CmR	pYMB025
pAE009	+130 *timP-3x flag*	CmR	pYMB025
pAE010	ΔuSL1 *timP-3x flag*	CmR	pYMB025
pAE011	ΔSL2 *timP-3x flag*	CmR	pYMB025
pAE012	ΔSL3 *timP-3x flag*	CmR	pYMB025
pAE024	M2 *timP-3x flag*	CmR	pYMB025
pAE025	M2' *timP-3x flag*	CmR	pYMB025
pAE027	M2 + M2' *timP-3x flag*	CmR	pYMB025
pAE030	M2 *timP*	CmR	pYMB023
pAE031	M2' *timP*	CmR	pYMB023
pAE033	M2 + M2' *timP*	CmR	pYMB023
pAE039	wt SD::*cspE* SD	CmR	pYMB025
pAE040	M2' SD::*cspE* SD	CmR	pYMB025
pAE041	M2 + M2'::*cspE* SD	CmR	pYMB025
pEH795	M6 *timP* (1)	CmR	

Table S2. Oligonucleotides used in the study.

Oligo ID	Comments	Sequence (5' → 3')
EHO-690	Northern blot probe for 5S rRNA	TACGGCGTTTCACTTCTGAGTTCGG
EHO-1023	Rev for truncation constructs cloning (1)	TGGAGAAACAGTAGAGAGTTGCGA
EHO-1344	Northern blot probe for *timP* mRNA (1). Binds to the ORF.	ACACAACGTGCCAGAAACAAGAAGCACGACACAAAAGCAT
EHO-1419	Fwd for TimR *in vitro* transcription template (1)	gaaattaatacgactcactataggCTACGGTGTGCCTGCGTTGCTATGGCAACAAAGCCTGCTGGAAAGGC
EHO-1420	Rev for TimR *in vitro* transcription template (1)	CACAAAAAAACCGCTCAATTGAGCGGTTTTTTTGTGCTGGTCCGGTTCGCGGCCTTTCCAGCAGGCTTTGT
EHO-1421	Fwd for *timP in vitro* transcription template (1)	gaaattaatacgactcactataGGCCCTTTCCGCCGTC
EHO-1422	Rev for *timP in vitro* transcription template (1)	AAGAAAAGAAAGCCGCCCAACA
EHO-1661	Fwd for +48 *timP* cloning	P-AACCCATGGCCGTAAGCG
EHO-1662	Fwd for +87 *timP* cloning	P-TATCACGGCATTATCGCCAGCGGT
EHO-1663	Fwd for +130 *timP* cloning	P-CGCAAGGACCGCAACATGA
EHO-1666	Fwd for M1 *timP* cloning	TAACCCAAGGCCGTAAGCGCAGGCAC
EHO-1667	Rev for M1 *timP* cloning	CGGCCTTGGGTTATCCTCTCCTG
EHO-1841	Fwd for M6 TimR *in vitro* transcription template	gaaattaatacgactcactataggCATGCCAGTGCCTGCGTTGCTATGGCAACAAAGCCTGCTGGAAAGGC
EHO-1918	Fwd for +48 *timP in vitro* transcription template	gaaattaatacgactcactataggAACCCATGGCCGTAAGCG
EHO-1919	Fwd for +87 *timP in vitro* transcription template	gaaattaatacgactcactataggTATCACGGCATTATCGCCAGCGGT
EHO-1920	Fwd for +130 *timP in vitro* transcription template	gaaattaatacgactcactataggCGCAAGGACCGCAACATGA
EHO-1931	Fwd for M7' *timP* cloning	[Phos]CCTTTCCATAACCCATGGCCGTAAGCG
EHO-1932	Fwd for M7' *timP* cloning	[Phos]TGAAGCCAGCGCCCGTTT
EHO-1933	Fwd for M7 *timP* cloning	[Phos]GCCGTCTCGCAAACGGGC
EHO-1934	Rev for M7 *timP* cloning	[Phos]CCTCTCCGCCTGGAGAAACAGTAGAGAG
EHO-1937	Fwd for ΔSL4 *timP* cloning	[Phos]AACATGAAGGTACGATGC
EHO-1938	Rev for ΔSL4 *timP* cloning	[Phos]ATCACGGCAGCACC
EHO-1963	Fwd for ΔuSL1 *timP* cloning	[Phos]TTCAGGAGAGGATAACCC
EHO-1964	Rev for ΔuSL1 *timP* cloning	[Phos]GGAAAGGGCCTGGAG
EHO-1965	Fwd for ΔSL2 *timP* cloning	[Phos]TATCACGGCATTATCGC
EHO-1966	Rev for ΔSL2 *timP* cloning	[Phos]TACGGCCATGGGTTAT
EHO-1967	Fwd for ΔSL3 *timP* cloning	[Phos]GCGGTCTTCGCAAGG
EHO-1968	Rev for ΔSL3 *timP* cloning	[Phos]AGCAGGCACTACGGT
EHO-2160	Fwd for M2 *timP* cloning	[Phos]GCGCTGCGTTCAGGAGAGG

EHO-2161	Rev for M2 *timP* cloning	[Phos]CCGTTTGCGAGACGG
EHO-2162	Fwd for M2’ *timP* cloning	[Phos]GCATTATCCGCAGCGGTGCTG
EHO-2163	Rev for M2’ *timP* cloning	[Phos]CGTGATAGCAGGCACTACG
EHO-2502	Antisense oligo for RNase H assay	CACCGCTG

SI References

1. L. Andresen, Y. Martínez-Burgo, J. Nilsson Zangelin, A. Rizvanovic, E. Holmqvist, The Small Toxic Salmonella Protein TimP Targets the Cytoplasmic Membrane and Is Repressed by the Small RNA TimR. *mBio* **11**, 10.1128/mbio.01659-20 (2020).

2. L. M. Guzman, D. Belin, M. J. Carson, J. Beckwith, Tight regulation, modulation, and high-level expression by vectors containing the arabinose PBAD promoter. *Journal of Bacteriology* **177**, 4121–4130 (1995).
